# 631. Sedation-Driven Variability in Antibiotic Response of Stenotrophomonas maltophilia

**DOI:** 10.1093/ofid/ofaf695.198

**Published:** 2026-01-11

**Authors:** Elizabeth May, Rachel Gray, Alex Do, Ashlan J Kunz Coyne

**Affiliations:** University of Kentucky College of Pharmacy, Lexington, KY; University of Kentucky College of Pharmacy, Lexington, KY; University of Kentucky College of Pharmacy, Lexington, KY; University of Kentucky College of Pharmacy, Lexington, KY

## Abstract

**Background:**

*Stenotrophomonas maltophilia* is an opportunistic pathogen that disproportionately affects vulnerable patients in high-acuity settings. These individuals often receive sedation for pain, agitation, and ventilation. The interaction between sedatives and antibiotics against *S. maltophilia* is unclear. This study evaluated whether common sedatives alter antibiotic activity and growth suppression against *S. maltophilia.*

**Figure:**
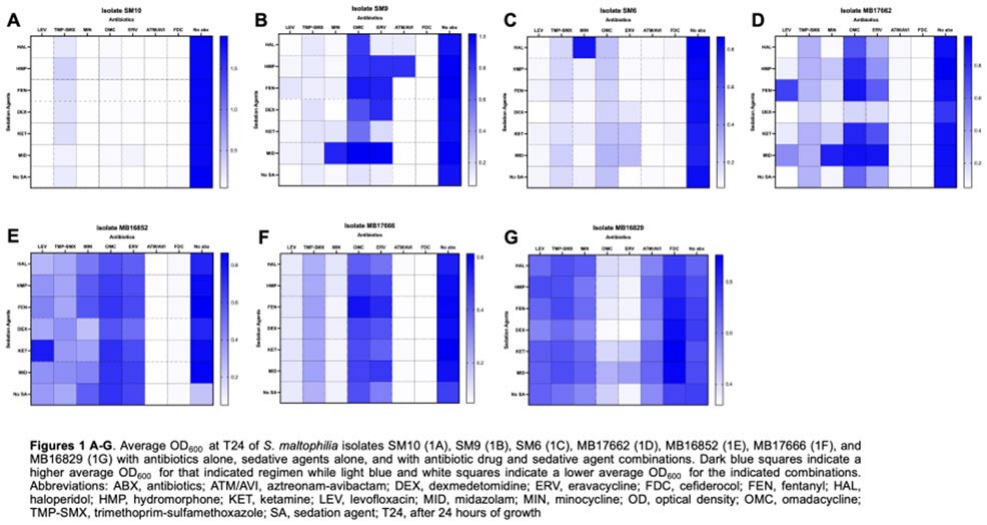

**Figure:**
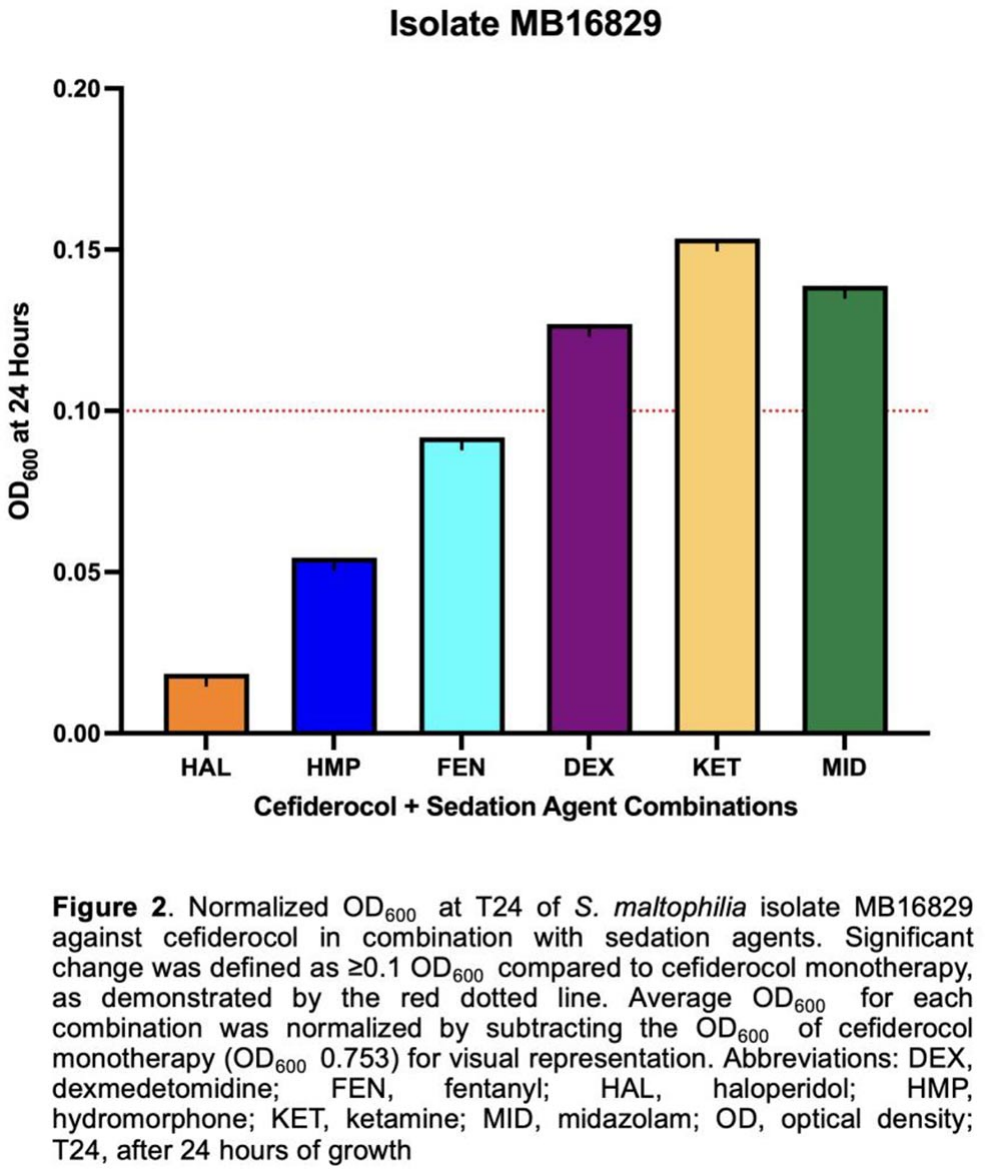

**Methods:**

Seven clinical *S. maltophilia* isolates (MB16829, MB16852, MB17662, MB17666 from hematologic malignancy patients at MD Anderson; SM6, SM9, SM10 from lung disease patients at UK Healthcare) were exposed to 8 antibiotics—levofloxacin (LEV), ciprofloxacin (CIP), trimethoprim/sulfamethoxazole (TMP/SMX), minocycline (MIN), omadacycline (OMC), eravacycline (ERV), aztreonam/avibactam (ATM/AVI), and cefiderocol (FDC)—alone or with haloperidol (HAL), hydromorphone (HMP), fentanyl (FEN), dexmedetomidine (DEX), ketamine (KET), or midazolam (MID). Growth (OD600) was measured every 15 minutes for 24 hours at 37°C. Significant changes were defined as ≥0.1 absorbance difference vs. antibiotic alone.

**Figure:**
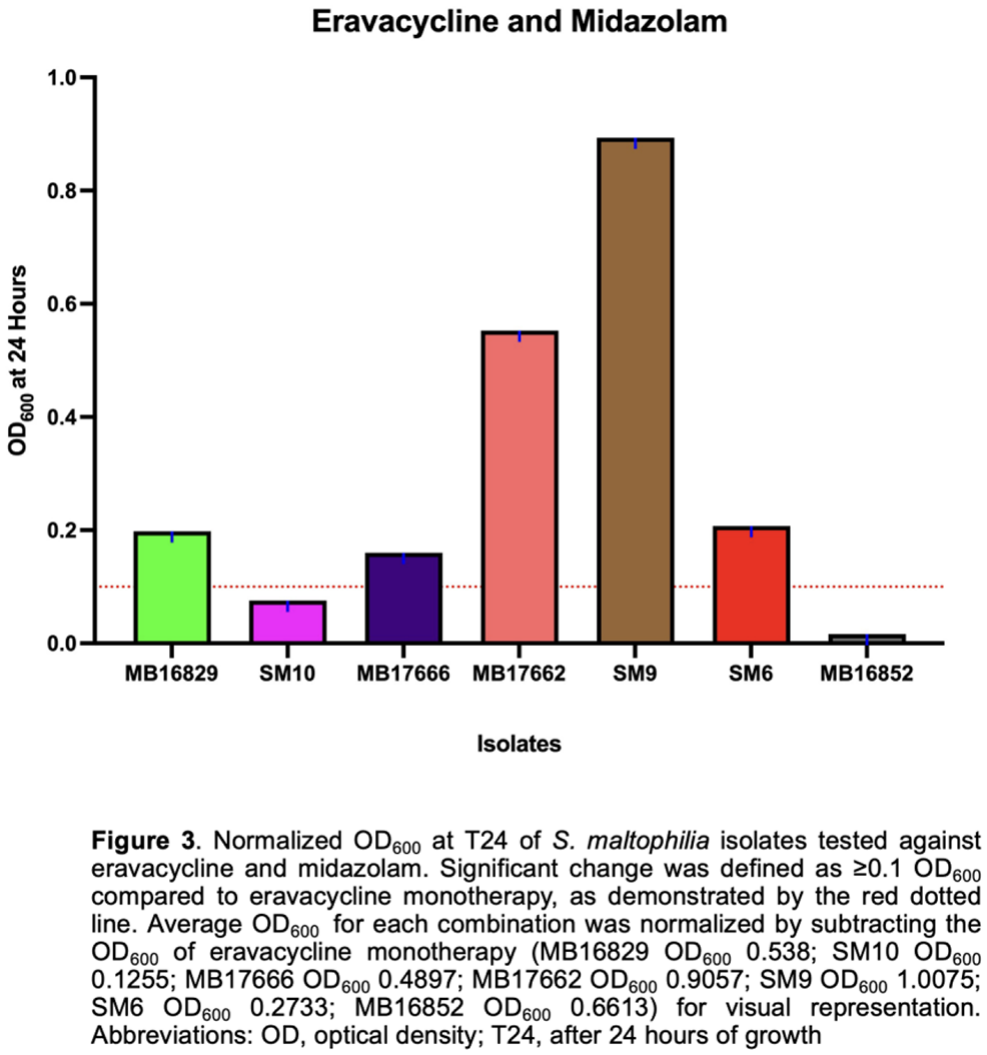

**Figure:**
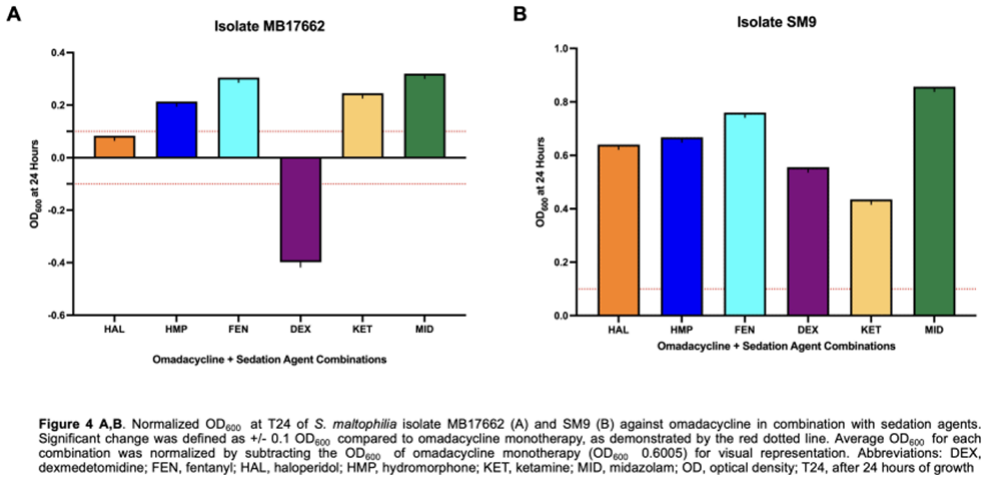

**Results:**

Sedative–antibiotic interactions altered *S. maltophilia* growth in 43 regimens (Figure 1). MID increased growth in 11 combinations, followed by FEN (10). DEX was the only sedative linked to decreased growth (6 regimens). ERV and OMC were most impacted, with 9 and 8 increased-growth combinations; 3 of 6 decreases also involved OMC. SM9 showed increased growth in 8 combinations, mainly with OMC and MID. Isolate 16852 showed decreased growth in 6 combinations—all with DEX. ATM/AVI and FDC remained stable, except in 16829, where 3 FDC combinations (+DEX, KET, MID) increased growth (Figure 2). ERV+MID increased growth in 17666 (0.538), 17662 (0.581), SM6 (0.418), and SM9 (1.016) vs. ERV alone (0.353, 0.353, 0.173, 0.114; Figure 3). In Figure 4, OMC combinations in 17662 (A) and SM9 (B) showed isolate-specific responses, with MID and FEN driving the greatest increases.

**Conclusion:**

MID and FEN most often increased *S. maltophilia* growth, especially with OMC and ERV in SM9 and 17662. DEX reduced growth in all six decreased regimens in 16852. FDC increased growth only in 16829. Findings highlight sedative effects on antibiotic activity in critical care.

**Disclosures:**

All Authors: No reported disclosures

